# Cell-free expression system: a promising platform for bacteriophage production and engineering

**DOI:** 10.1186/s12934-025-02661-9

**Published:** 2025-02-17

**Authors:** Hanzada Nour El-Din, Maryam Kettal, Serena Lam, José Granados Maciel, Danielle L. Peters, Wangxue Chen

**Affiliations:** 1 Human Health Therapeutics Research Center, National Research Council Canada, Ottawa, ON K1N 5A2 Canada; 2 Department of Biology, Brock University, St. Catharines, ON Canada

**Keywords:** Cell-free expression system, Bacteriophages, Transcription-translation, Phage engineering, Antimicrobial resistance

## Abstract

Cell-free expression is a technique used to synthesize proteins without utilising living cells. This technique relies mainly on the cellular machinery —ribosomes, enzymes, and other components — extracted from cells to produce proteins in vitro. Thus far, cell-free expression systems have been used for an array of biologically important purposes, such as studying protein functions and interactions, designing synthetic pathways, and producing novel proteins and enzymes. In this review article, we aim to provide bacteriophage (phage) researchers with an understanding of the cell-free expression process and the potential it holds to accelerate phage production and engineering for phage therapy and other applications. Throughout the review, we summarize the system’s main steps and components, both generally and particularly for the self-assembly and engineering of phages and discuss their potential optimization for better protein and phage production. Cell-free expression systems have the potential to serve as a platform for the biosynthetic production of personalized phage therapeutics. This is an area of in vitro biosynthesis that is becoming increasingly attractive, given the current high interest in phages and their promising potential role in the fight against antimicrobial resistant infections.

## Cell-free expression system: introduction

Cell-free expression systems (CFES), also referred to as cell-free protein synthesis (CFPS) and cell-free gene expression (CFGE), has emerged as a powerful tool to complement or replace traditional in vivo expression (will be used in this review to refer to cell-based expression) techniques owing to the unique advantages that the system offers for molecular biology and biotechnology purposes. Indeed, since its initial emergence in the 1960s, this system has revolutionized the world of research and biomanufacturing by providing versatile platforms for protein synthesis, enzyme engineering, synthetic biology applications and, more recently, bacteriophage (phage) production and engineering.

### Background on cell-free expression systems

Traditional in vivo protein production is reliant on the production host; thus, the growth environment and physiology of the host must be considered and optimized. The use of a production host requires long experimental hours as the hosts’ growth is a rate limiting step. Further, in every production cycle, the cells must be harvested, the proteins purified, and appropriate biological assays conducted to determine the quality and activity of the product. That being said, there is a pressing need for alternative production platforms that are more controlled, not only to elevate the ease of the production, but also to meet the Good Manufacturing Practice guidelines that are particularly challenging when working with biological entities.

CFES was initially utilized in 1961 by Nirenberg and Matthaei as a controlled platform to study the mechanism of synthesis of template or “messenger” RNA and the enzymes involved in this process [1]. Moving forward with the same system, the genetic code was deciphered three years later [2]. What started as an empirical experimentational system was transformed to a promising bioengineering and biomanufacturing platform in the 21st century with the development of robust protocols and well-studied components, such as the S30 extract protocol [3], cytoplasm supplementation (e.g.; ions and PEG8000), and the purified reconstituted system (PURE), which have positioned CFES on a solid foundation in the field of biomanufacturing [4, 5]. The system has been used to express various proteins, including peptide therapeutics such as chimeric endolysins [6], cecropin [7] and beta-defensin-2 [8] and difficult-to-express cytotoxic proteins as onconase [9]. Interestingly, the CFES was successfully used, with some modifications, for membrane protein synthesis, such as G protein-coupled receptors [10] and phospho-N-acetyl-muramoyl-pentapeptide translocase, MraY, involved in the synthesis of the peptidoglycan layer in *Bacillus subtilis* [11].

The first step in creating cell-free systems is to grow and collect cells from the chosen host to produce the reaction’s chassis. Most systems use prokaryotic organisms, specifically *Escherichia coli* as it is a thoroughly studied organism and can be well controlled in an experimental context [12–14]. Eukaryotic systems are also explored in CFES to facilitate post-translational modifications that are challenging to replicate in prokaryotic systems. For example, yeast and insect cell extracts have been used to express glycosylated or membrane-bound proteins for functional studies, although these systems often require more intricate optimization and are generally more costly [15]. The preparation of cell extracts can be scaled up for industrial settings with lower associated costs. They also offer the advent of being multi-component metabolic systems [16]. To obtain a cell extract of high quality and high protein content, an enriched medium is often used with most protocols. The cells are then lysed to acquire the extract of the cellular components. This extract contains all the molecules, proteins, and organelles of a living and active cell (ribosomes, RNA polymerase, etc.), and can be directly used, or undergo further preparatory steps, depending on the application [4, 16]. The post-lysis steps include clarification of the lysate through centrifugation, a runoff reaction, and dialysis, then storage, generally at – 80 °C. Thereafter, these extracts can be used at one’s convenience by adding linear or plasmid DNA templates, dNTPs, amino acids and other molecular reagents to mimic the cytoplasmic crowding and enhance the reaction mix to obtain the desired products [17, 18].

CFES have numerous applications depending on the components integrated into the system. The most common use is protein synthesis or “cell-free transcription-translation” (TX-TL), which involves the insertion of the gene of interest (GOI), tagged to allow for downstream purification. Depending on the protein expressed and the aim of the study, it is an optional step to provide a reporter fluorescent protein via plasmid or linear DNA to allow for timely monitoring of expression. The most common protein expression system use the phage T7 RNA polymerase to drive transcription of the GOI from the T7 promoter, and protein production from the resulting mRNA occurs with *E. coli* translation machinery [19]. Further CFES development has led to the creation of the PURE (protein synthesis using recombinant elements) system, which operates in a fully purified cell-free environment with a simple biochemical background based on T7 machinery. Unfortunately, the PURE system is costly when compared to traditional lysate-based TX-TL systems [4, 20–22].

### CFES for phage production

A rise in antimicrobial resistant bacteria has rendered many antibiotics ineffective, leading to an urgent need to find viable alternatives to treat these infections [23]. One of the promising alternatives is the use of phages as they possess several amiable characteristics. For instance, phages are known for their specificity and propagation capability at the site of infection, which prolongates their effect with less need for repeated treatments. On another front, phages can be engineered to enhance their favorable features (e.g., lytic ability and host range tropism) and lessen the undesirable ones (e.g., integration into bacterial hosts and immunogenicity to humans) [23, 24].

However, phage production is a challenging process with several rate-limiting steps. Among those challenges comes the long propagation time that can take up to several days and is affected by the culture media and growth kinetics of the propagation host. Additionally, the host needs to be non-pathogenic, well-characterized, and devoid of encoded prophages that can cause lysogenic phage contaminations [25]. Once propagated, endotoxins must be removed to an established level to allow for the therapeutic use of the phages. Depending on the phage undergoing purification, endotoxin removal can have differing efficiency and usually results in a significant loss in titer, as well as changes in phage infectivity [26]. Further, there is a lack of Good Manufacturing Process facilities which are capable of producing phage lysate using Risk Group 2 pathogens. Optimization of phages with in vivo engineering can take even longer with the unpredictable licensing process of genetically modified organisms and it is not feasible with all phages and bacterial hosts [24, 27]. Phage production using CFES is a viable solution to reduce production time and provide greater control over the entire reaction process, thereby offering a flexible, modular platform [28].

### Research gaps and goal of the review

Despite the alluring traits of the CFES, there are still many areas for optimization and improvement before this platform can be used for routine phage production. The first documented use of CFES for production of functional phages used cell extracts from *E. coli* with the coliphages MS2, ΦX174, T7 and T4 [29, 30]. The coliphages used in this system infect the *E. coli* strain used to produce the cell-free extract. Recent work has demonstrated the feasibility of the *E. coli-*based CFES to produce phages targeting several members of the Enterobacterales order, and with phage rebooting via co-expression of the appropriate host factors to produce phages targeting bacteria distant from *E. coli*. Moving forward with the system, cell lysates and reaction mixtures have yet to be optimized to enhance the production yields and to allow for the production of phages with DNA modifications, as well as engineered phages.

From another perspective, the cell-free extract preparation in phage directed studies is mostly dependent on *E. coli*. Being a Gram-negative bacteria, endotoxins (i.e. lipopolysaccharides) are an integral part of its structure and are released during the cell lysis process [31]. The endotoxin level from *E. coli* cell extracts was found to be higher than when other model organisms were used as a chassis for the extract preparation [32]. Still, the *E. coli*-based system exhibits superior expression. This is an area that needs to be further investigated in cell-free expression phage directed studies.

**Fig. 1:**
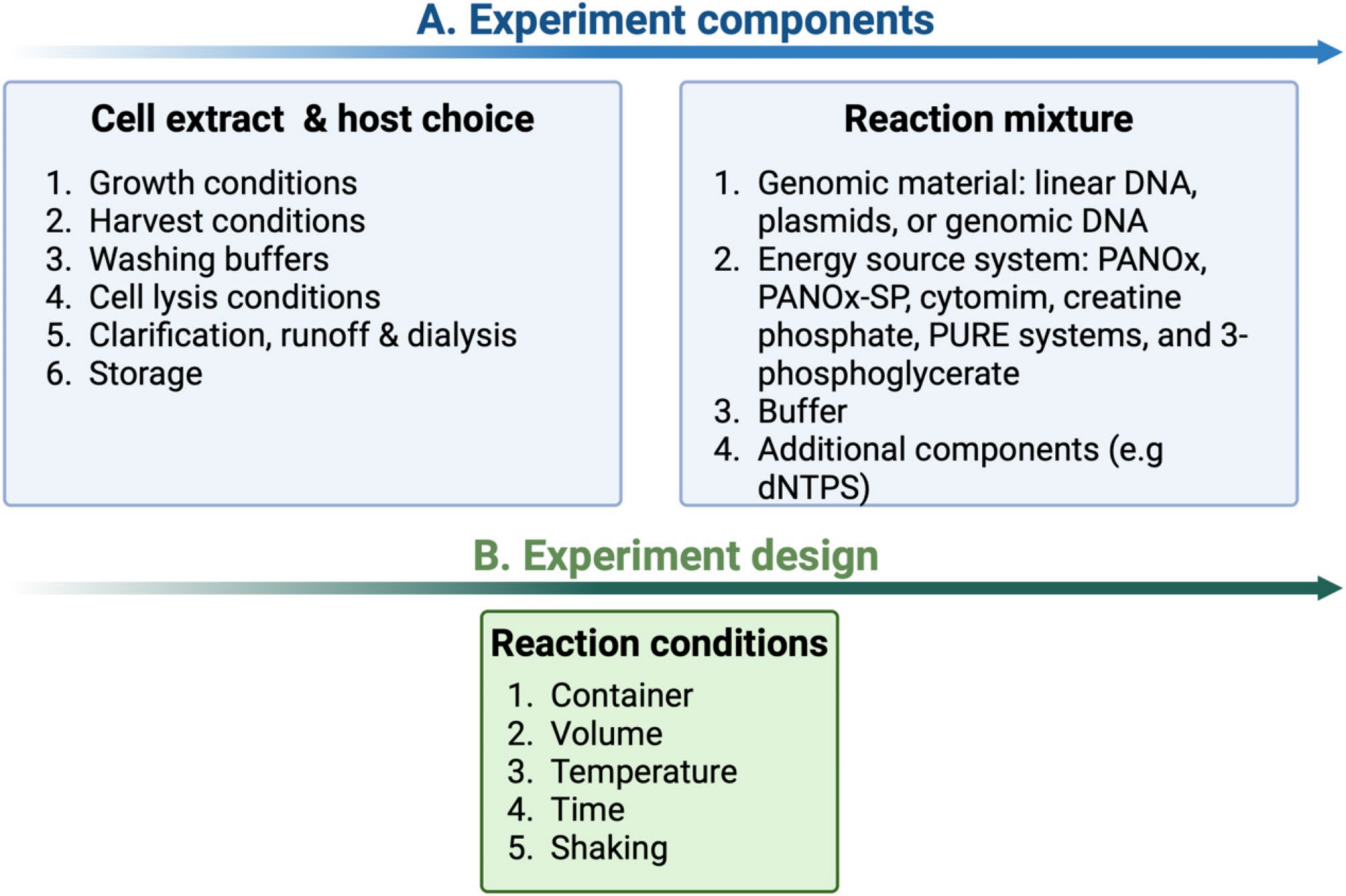
Overview of the cell-free expression system workflow. A diagram showing the key points addressed throughout the review article in terms of (**A**) cell-free expression experiment components and (**B**) experiment design. Figure is generated using BioRender

The CFES is made up of three components which can be optimized: cell extract, reaction mixture and DNA templates. Further, reaction conditions can be manipulated to decrease the time needed to prepare the cell extract and reaction mixture, while also decreasing associated costs. In the following sections, we will discuss the optimization of each of the system’s components for protein production generally, and phage specifically, based on published literature and studies **(**Fig. 1**)**. Finally, we will present our perspective on this system’s future development to better fulfil its role in phage production, while emphasising the areas that warrant additional research as well as the system’s limitations.

## Cell extract preparation

**Fig. 2:**
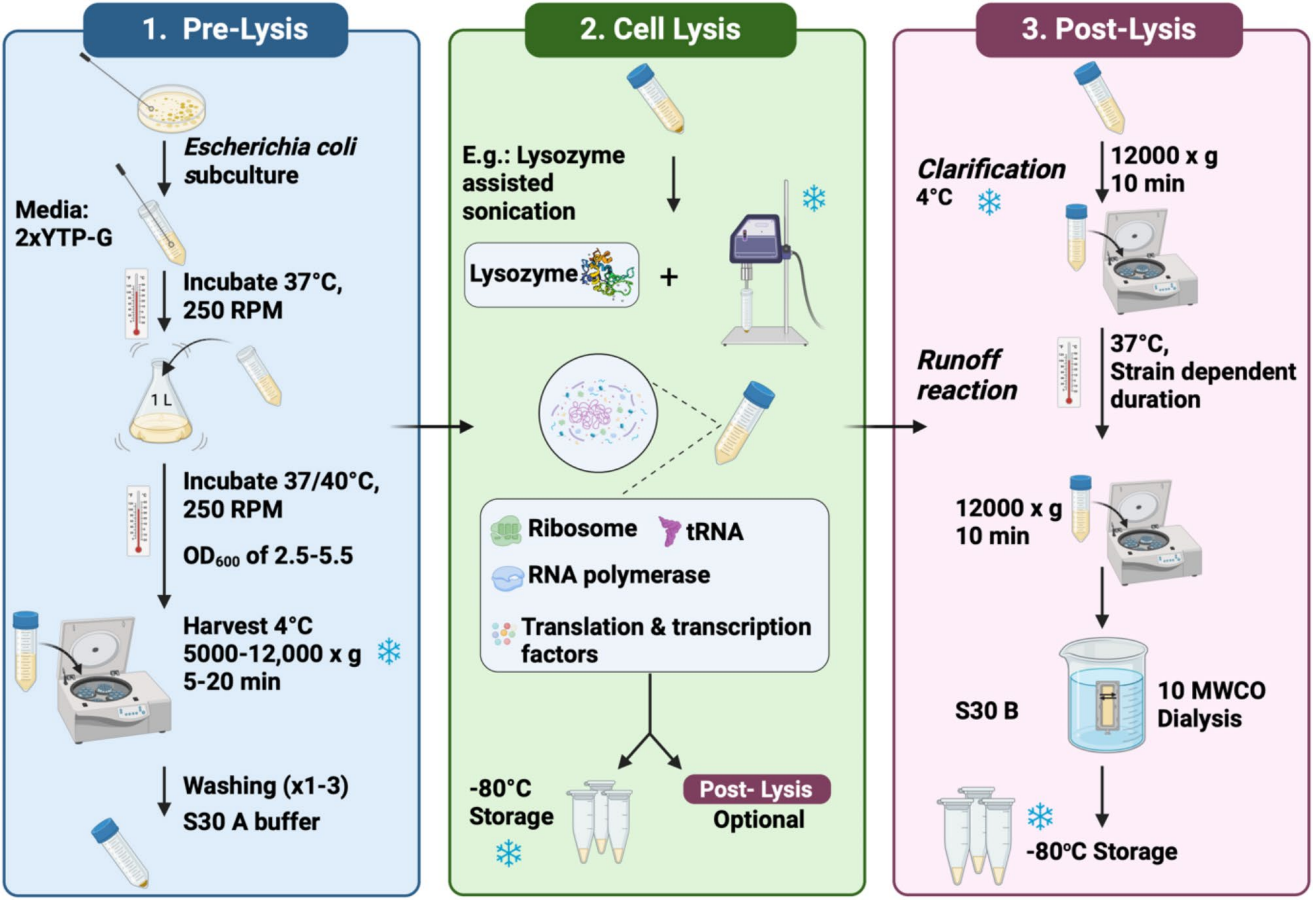
Overview of the fundamental procedure used to generate *E. coli* lysate for phage directed CFES. The conditions shown are those used for *E. coli* given that it is the only organism used so far in phage production. ***(1) Pre-lysis step***: which entails streaking of the organism from stock, pre-culturing in yeast extract tryptone/phosphates glucose media (2xYTP-G), culturing, harvesting and finally washing with S30A buffer. ***(2) Cell lysis step***: this step involves lysing the bacterium to release the cellular machinery. The lysozyme assisted sonication method is highlighted. ***(3) Post-lysis step***: final processing of the extract involves centrifugation, a runoff reaction, then dialysis with the S30B buffer, and storage for long term usage. Figure is generated using BioRender

Preparing a cell extract for cell-free expression system entails several steps **(**Fig. 2**)** that shall be planned based on the expression target.

### Host growth conditions

#### Culture media and conditions

The growth conditions of the bacterial host used for producing cell extracts are critical for maximizing protein production, which is essential for efficient transcription and translation. The critical consideration in preparing the cell-free extract is to obtain healthy cells, which is not solely defined by achieving a high cell mass. The host organism’s growth rate is also pivotal, as high cell mass achieved through slower growth rates results in a lower concentration of essential translational machinery. Faster-growing cells contain more ribosomes per unit cell mass, which is needed for efficient translation [33]. Typically, 2x yeast extract tryptone (2xYT) media composed of tryptone, yeast extract, and sodium chloride is chosen as the growth medium as it is nutrient dense [34]. Phosphate supplementation in the media is necessary to stabilize the pH and to reduce the phosphatase activity in the growing bacteria [12, 35]. The growth media can also be supplemented with an additional carbon source such as glucose, ribose, or maltodextrin. The purpose of the sugar is to provide the cells with sufficient materials to generate energy, enhancing ATP production and regeneration [15, 36–44]. An exception to the streamlined 2xYTP-G media usage was done in Doi and Fujiwara study as they used Lysogeny broth (LB) for culturing *E. coli* BL21(DE3) codon plus (RIL) [45]. In systems where a BL21 (DE3) strain is used, it was proved that adding isopropyl β-D-1-thiogalactopyranoside (IPTG) during the mid-log phase of bacterial growth increases the production of the encoded T7 RNA polymerase that is under the control of the *lac*UV5 promoter [38, 39, 41, 43]. By expressing T7 RNA polymerase during cell growth, the expense and complexity of additives required for CFPS processes are reduced, as this protein will be readily available, in a high concentration, to transcribe the DNA template in the CFE reaction [38]. A 2018 study on cell extract optimization determined the optimal induction for T7 polymerase expression and growth times in 150 mL cultures to be 216 and 276 min, respectively. These times corresponded to 28 and 58.5% of the total cell growth period [38]. The temperature used for host growth depends on several factors, including the type of host used for cell extract preparation, the culture media, and the type of DNA template used in the CFES. In this review, we will focus on the optimal conditions for prokaryotic hosts, specifically *E. coli*, as it is the only host studied for creating cell-free phage production systems. Although 37 °C is the standard temperature for growing *E. coli*, cell extracts produced at 30 °C, when combined with a linear DNA template, yielded three times more protein than those prepared at 37 °C. This increase was attributed to a reduction in RecD protein in the 30 °C extracts, which reduced degradation of the linear DNA template in the CFES [46].

In Noireaux’s “All-*E. coli* TX-TL toolbox 3.0”, two modifications enabled protein synthesis to reach levels above 3 mg/ml. One modification involved growing CFES cells at 40 °C instead of 37 °C [44]. This adjustment was based on a 2005 study comparing two growth temperatures and two types of media: one medium contained 2 g/L yeast extracts as per Pratt [47], while the other media, referred to as the aa-enriched medium, was further supplemented with casamino acids and three amino acids (asparagine, glutamine, and tryptophan) [48]. Both culture media were tested at 37 and 42 °C. In the aa-enriched medium, bacterial growth rate significantly increased at 42 °C, resulting in a more active S30 extract [48].

Mostly, an agitation speed of 200 to 250 rpm for cell growth is used. However, no studies have specifically investigated the effect of rpm on cell extract preparation for subsequent testing in CFES. Baffled bottom flasks are typically used to improve aeration, thereby reducing oxygen-related stress that could otherwise cause harmful metabolic changes under these conditions.

#### Harvest conditions

The translation machinery is most active during the mid-exponential growth phase. Hence, it is the growth phase of choice for harvesting cells [24, 28, 32, 34, 36, 37, 40, 41, 49–54]. In 2015, Jewett and Kwon investigated the optimal harvest times for different *E. coli* strains, specifically comparing the BL21 Star (DE3) and the C495 strain, a recoded genomic derivative of K12 MG1655 (strain C49548) [36]. The researchers found that CFPS performance was consistent when the BL21 Star (DE3) strain was harvested at an OD_600_ between 2.5 and 5.5, while the C495 strain produced the most active extracts at an OD600 between 2.5 and 3.5 [36].

For cell harvesting, it is known that high centrifugal force can stress bacterial cells and should therefore be considered during optimization [55]. Typically, centrifugation is performed at speeds of 5,000 x g to 10,000 x g at 4 °C for 5 to 20 min [12, 28, 37, 38]. In a 2016 study, Doi and Fujiwara compared centrifugation speeds of 12,000 x g, 16,000 x g, and 25,000 x g, adjusting centrifugation times to achieve the same total centrifugation force (rcf × time) as compared to 25,000 × g for 60 min. Their results showed that the protein yields were similar for the three tested centrifugation conditions [45]. The same conclusion was drawn from the study conducted by Jewett and Kwon as they tested five centrifugation speeds (10, 12, 15, 18 and 21 x g). The activity for cell extract was not significantly different at speeds higher than 10,000 x g [36].

#### Washing buffer

S30A buffer is the commonly used washing buffer for the system and it is typically composed of 50 mM Tris, 14 mM magnesium, 60 mM potassium, and 2 mM dithiothreitol (DTT) at pH 7.7 [12, 15, 32, 34, 43, 50–54]. The magnesium and potassium salts vary, with earlier studies favouring acetate forms and more recent studies using glutamate forms. Both glutamate and acetate act as buffering agents to stabilize pH in the CFES, but glutamate offers higher buffering capacity across a broader pH range, which can enhance protein stability and activity. Variations in S30A buffer composition include lower Tris concentration (10 mM) with a slightly higher pH of 8.2 [28, 36, 39–41]. Further alterations to the S30A buffer include the use of 6 mM β-mercaptoethanol instead of DTT [37]. While both β-mercaptoethanol and DTT reduce disulfide bonds, β-mercaptoethanol additionally protects sulfhydryl groups from oxidation, which can be advantageous depending on the CFES objectives.

The reported number of washes ranges from one to three although most laboratories use three washes. A 2005 study found that one, two, or three washes had no significant impact on extract productivity, suggesting that extract performance is unaffected by wash frequency [56].

### Cell lysis

**Fig. 3:**
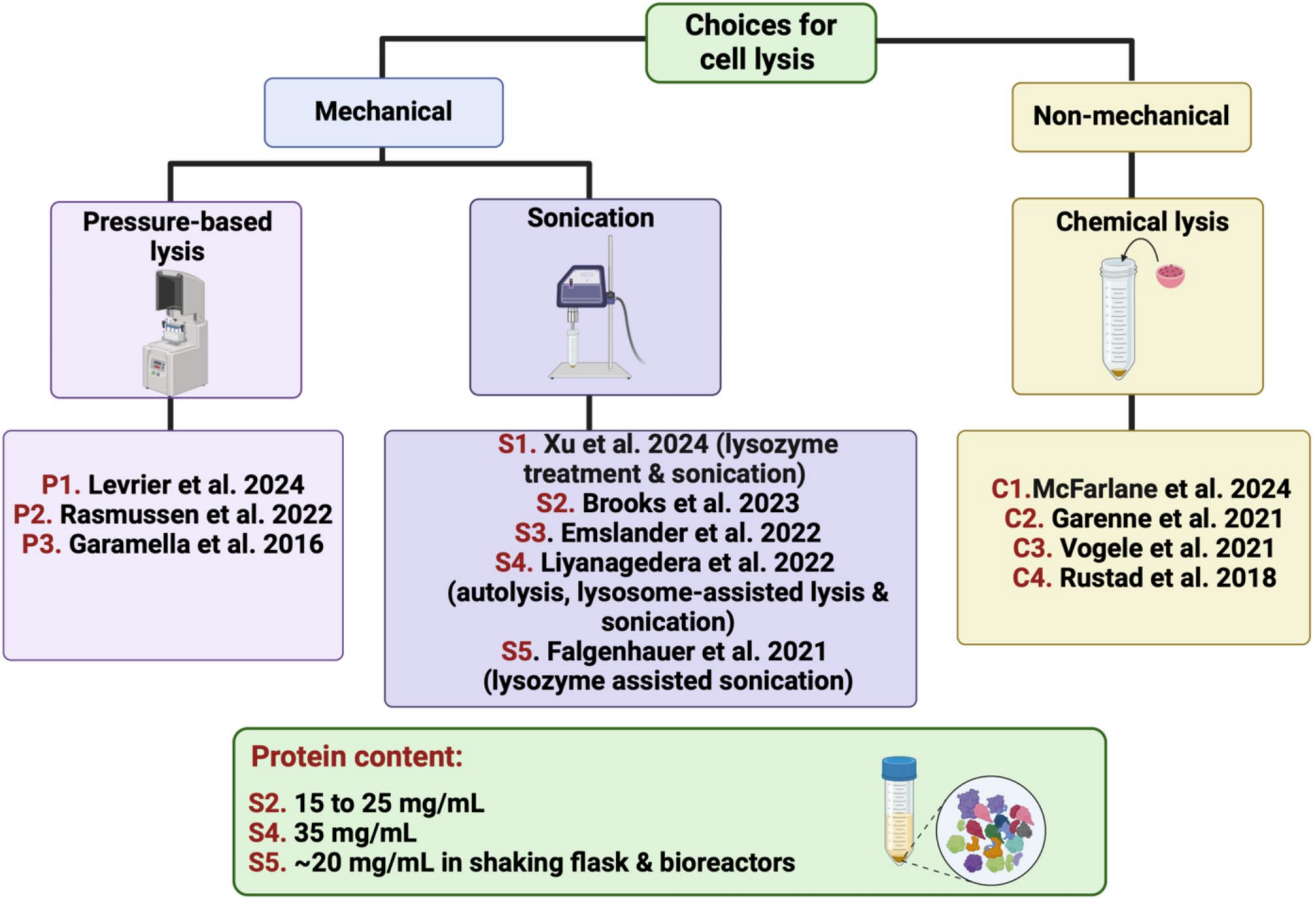
Overview of cell lysis techniques used in phage directed CFES studies. The figure shows the two reported cell lysis approaches, either mechanical or non-mechanical. Under each approach the used lysis methods are mentioned along with the reference studies. The protein content of the cell extract is indicated when available. Figure is generated using BioRender

Effective lysis techniques are essential for creating cell extracts for CFES, as they enable efficient extraction of functional components such as enzymes, ribosomes, and other cellular machinery. The goal is to rupture cells while preserving the functionality of these intracellular constituents. Several lysis methods are commonly used, as summarized in the following section. Techniques specifically applied to phage production are shown in Fig. 3.

#### Pressure-based lysis

Pressure-based cell lysis systems, also known as mechanical lysis, are commonly used in CFES studies for large-scale cell extract preparation due to their scalability. Typically, cell presses apply pressures up to 30,000 pounds per square inch (PSI) to lyse cells, such as the French press [15, 19, 37, 38, 49]. More recent studies, however, have used lower pressures, between 10,000 and 15,000 PSI to produce extracts from various microorganisms, including *E. coli*, *Bacillus subtilis*, *Corynebacterium glutamate*, and *Vibrio natriegens* [32, 51]. In Noireaux et al. study published in 2024, they utilized cell press approach to prepare cell extracts for phage production, achieving titers up to 10^11^ plaque forming units (PFU) per mL after a 3-h incubation [57]. This titer falls in the high end when expressing phages using the cell-free system which is further demonstrating the effectiveness of this lysis method.

Another aspect to be considered when using homogenization is the flow rate. To that end, Swartz and Alexei investigated high-pressure homogenization with an emphasis on flow rate, comparing slow (0.25–0.4 mL/min), medium (1.2–2.0 mL/min), and fast (2.8–7 mL/min) flow rates [58]. The rationale for testing higher flow rates was to shear genomic DNA (gDNA), minimizing its presence in later stages. Residual gDNA can negatively impact CFES by potentially triggering the synthesis of unintended proteins. Results showed that all flow rates, up to 7.0 mL/min, prevented background synthesis in a negative control reaction, and there was no loss of extract activity, supporting the robustness of high-flow-rate homogenization for cell extract preparation [58].

#### Non-pressure based lysis

Non-pressure-based lysis techniques include bead milling, bead vortexing, and bead beating, which utilize ceramic or glass beads to lyse cells [42]. In 2010, Noireaux and his team employed 0.1 mm diameter glass beads with a mini bead-beater to lyse cells from a 2 L cell culture, producing 6 mL of crude extract with a final protein concentration of 27 to 30 mg/mL. Notably, the cell extract retained its activity after thawing [34]. In 2013, the widely cited protocol by Sun et al. also used similar sized beads, vortexing samples for 30 s while keeping them on ice to prevent heat-induced loss of activity [12]. Compared to sonication and French press methods, bead beating is cost-effective and improves reproducibility.

#### Sonication

Sonication uses ultrasonic energy to disrupt cell membranes, leading to cell lysis. However, two major concerns arise with this method: the heat generated can denature proteins within the extract, thereby reducing system efficiency, and insufficient energy may result in incomplete lysis, yielding extracts with lower total protein concentrations [42]. Additionally, repeated sonication-cooling cycles can introduce heat shock, potentially deactivating catalysts in the extract.

To address these issues, samples are placed on ice, and sonication is performed in well-studied bursts of on and off cycles to prevent overheating and heat shock. This is achieved by considering the optimal energy input based on the extract’s volume [36]. Jewett and Kwon utilized a Q125 Sonicator with a 3.175 mm diameter probe set at 20 kHz frequency and 50% amplitude, applying 10 s on and off bursts. They reported optimal energy inputs of 556 Joules (J) and 309 J for 1.5 and 0.5 mL sample volumes, respectively, when preparing extracts from either BL21 Star (DE3) or C495 *E. coli* strains [36]. Based on the linear trends for total energy inputs covering the highest CFPS activity, an equation that can be used to calculate the optimal needed energy, in joules, based on the volume was concluded: For BL21 Star™ (DE3): [Energy] = [Volume(µL)-33.6]·1.8^− 1^ [36].

#### Freeze-thaw

The freeze-thaw method for cell lysis utilizes the sharp ice crystals formed during freezing to disrupt cell membranes. Although not widely used, groups often rely on using it concurrently with another lysis approach or under specific experimental circumstance [45, 59]. In 2017, Hasty et al. group demonstrated a reproducible and cost-effective approach by combining programmed cellular autolysis with freeze-thaw or freeze-dry cycles for the rapid production of cell-free lysates. A genetically modified *E. coli* BL21-Gold (DE3) strain carrying the pAD-LyseR plasmid, which enabled the production of the Lambda phage endolysin protein R, was used in this study. Protein R degraded the bacterial cell wall, increasing the sample’s susceptibility to freeze-thaw cycles. The resulting cell-free extract proved highly efficient and compatible with linear DNA templates [59].

#### Chemical lysis

Chemical lysis, including enzyme and detergent use, disrupts the cell walls to achieve lysis. Lysozyme is commonly used for enzymatic disruption by degrading the peptidoglycan layer of the bacteria, resulting in an unstable cell structure. Detergents such as Tween 20, Triton X, and radioimmunoprecipitation assay buffer are also used [42]. This approach is often accompanied by a second lysis method in parallel or sequentially.

In 2016, Doi and Fujiwara introduced a method termed LoFT, combining lysozyme treatment, osmotic shock, and freeze-thaw cycles to prepare cell extracts [45]. They concluded that increasing freeze-thaw cycles reduced the supernatant volume without affecting protein yield. Thus, one freeze-thaw cycle using liquid nitrogen is sufficient to produce osmotic shock and subsequent cell lysis. Although this method resulted in lower protein yield, it does have its advantages. The approach can be done on smaller volumes of culture, performed in parallel, and does not rely on specialized equipment for success. Additionally, the materials used are relatively cheap and readily available in most laboratories [45].

In 2021, Falgenhauer et al. explored lysozyme-assisted sonication (LAS), hypothesizing that lysozyme could weaken the integrity of cell walls and support the sonication‐induced lysis. The effects of sonication cycle number and lysozyme concentration on the total protein yield in the cell-free extract and expression of a fluorescent protein in the CFES was assessed [53]. Without lysozyme, five sonication cycles produced the lowest mean protein content (~ 6 mg/mL), determined by bicinchoninic acid assay, and generated the lowest fluorescence signal intensity. Increasing sonication cycles to 15 tripled protein content and fluorescent production in the CFES [53]. Samples treated with lysozyme (0.5 or 1 mg/mL) without sonication also showed improved protein content and fluorescence compared to sonication alone. However, the combined LAS approach did not enhance lysis efficiency as measured by protein content but doubled fluorescent protein expression in the CFES. The study established an optimal protocol using five sonication rounds at 30 kHz and 10% amplitude with 0.5 mg/mL lysozyme, achieving phage T7 titres of 10^8^ PFU/mL, surpassing a commercial kit by an order of magnitude [53].

Notably, optimal sonication cycles vary with cultivation methods, requiring fewer cycles in shaking flasks than in bioreactors. This difference is attributed to the altered morphology of bacteria grown in bioreactors under high shear forces, which likely increases tolerance to more sonication cycles [60].

Directly after lysis, it is recommended to supplement additional DTT (2–3 mM) to the cell lysate and invert it several times for proper mixing before moving ahead with the following steps [41, 61].

### Post lysis

#### Clarification

Following cell lysis, a clarification step is needed to remove insoluble material and debris, isolating the enzymes, small molecules, and co-factors needed for the CFES [42]. Crude extract of the cells can be directly used in the CFES; however, due to the viscosity of the extract at this stage, it is hard to handle in the subsequent steps. Further, high levels of background expression are observed if a clarification step is not incorporated [62].

In 2006, Kim and his group introduced a simplified clarification method, centrifuging crude lysates at 12,000 x g for 10 min, followed by a brief incubation at 37 °C [62]. This method, effective for certain *E. coli* strains (Rosetta, BL21, and BL21-Star), reduced preparation costs by 80% while increasing productivity by 1.5 times. In 2010, Noireaux and Shin applied a two-step clarification process with centrifugation at 30,000 x g for 25 min pre-runoff reaction, followed by a second 30,000 x g spin for 10 min to remove degraded DNA [34].

Building on this, the Sun et al. protocol involved a clarification spin at 12,000 x g for 10 min at 4 °C, and a post-runoff spin at 12,000 x g for 10 min at 4 °C, which achieved reliable protein production despite the lower centrifugation speeds [12]. In 2015, Chan et al. showed that centrifugation speeds above 10,000 x g at 4 °C for 10 min effectively removed unlysed cells without significantly impacting extract activity [36]. Similarly, a single 30,000 x g centrifugation step achieved a protein concentration of 28 mg/mL [49], while Swartz found that omitting a second centrifugation step decreased expression efficiency [56].

The ideal clarification protocol varies with the strain and target proteins. For best results, a high-speed centrifugation (e.g., 12,000 x g or 18,000 x g) followed by a lower-speed spin post-runoff (e.g., 12,000 x g or 10,000 x g) is a common practice and can be optimized as needed.

#### Runoff reaction

Following cell extract clarification, a runoff reaction degrades endogenous DNA and mRNA via exonucleases, facilitating the dissociation of mRNA from ribosomes [37, 63]. However, proteins were also successfully expressed without the runoff reaction [38, 43]. The most common temperature and duration for incubation of a runoff reaction is 37 °C for 80 min. These conditions enabled thawing the cell extract once prior to use without losing its activity [19, 34, 44]. Runoff reaction was also done using the same conditions but with shaking between 200 and 250 rpm [12, 36].

Runoff incubation time can vary with bacterial strains: while prolonged times negatively impacted CFPS activity in BL21 Star (DE3) extracts, they enhanced expression capabilities in strain C495 [36]. Alternatively, higher incubation temperatures (e.g., 42 °C for 45 min) have also been effective for generating extracts with robust protein expression profiles [37].

The sequential process of clarification followed by runoff was proved essential for an active cell extract production. It has been demonstrated that performing the runoff reaction before centrifugation leads to a dramatic loss of extract activity [58]. A final 10,000 x g centrifugation at 4 °C for 10 min is standard post-runoff to remove precipitates formed during the process.

#### Dialysis

A dialysis step can be done before storage to remove small molecules that could disrupt transcription and translation [12]. This is an optional step, depending on the intended application of the cell-free system [41, 61]. Dialysis is commonly done using a dialysis membrane, tube, or cassette with a 10 kDa molecular cut off against S30B buffer composed of 5 mM Tris, 14 mM magnesium, 60 mM potassium, and 1 mM DTT at pH 8.2 [12, 34, 37, 50]. Deviations from this standard recipe include higher potassium (150 mM) [24, 49], adjusted Tris (10 mM or 2 mM) [37, 49], and reduced DTT (0.5 mM) [37].

### Storage

Storage methods for crude extracts vary depending on intended applications, such as laboratory, industrial, or point-of-care use like pharmacy-on-a-chip microfluidic devices. Lyophilization, for example, enables extracts to be stored at room temperature for 60 days, making it a cost-effective, portable option for protein production [64]. The most common method, however, is flash-freezing in liquid nitrogen and storing at − 80 °C [12, 38, 42], though some studies skip the flash-freeze step [19, 34, 57]. For portable uses, freeze drying extracts onto paper discs and other porous materials has shown promising results, maintaining protein expression after rehydration [65]. These findings may pave the way for usage of CFES in further applications, particularly in diagnostics.

## Host strains

In developing CFES, the selection of an appropriate host strain is crucial and should align with the study’s specific objectives and required expression efficiency. The majority of extracts produced for CFE use prokaryotic organisms which are mostly genotypically modified to impart distinct properties tailored for specific applications.

Generally, *E. coli* remains the predominant choice for CFES, benefitting from decades of research that have yielded optimized protocols and genetically modified strains to suit various applications [42]. The most used strains are BL21-Rosetta and BL21, with and without DE3 modifications. The versatility of *E. coli* is well documented, and it serves as an effective model for producing proteins, especially when genetic manipulations are used to expand the incorporation of noncanonical amino acids, enabling the synthesis of proteins with novel functionalities beyond the natural 20 amino acids. This attribute is particularly useful for engineering diverse macromolecules with unique properties [42].

Advances in CFES have opened up opportunities for alternative prokaryotic hosts. In 2021, *B. subtilis*, *C. glutamate*, and *V. natriegens* were investigated as potential CFES chassis, each offering unique advantages for industrial applications [32]. For instance, *V. natriegens*, known for its rapid growth rate, and *B. subtilis*, a robust industrial organism, may provide valuable alternatives to *E. coli* for large-scale applications [32]. A primary benefit observed in these alternative hosts is the significantly lower endotoxin levels in their cell extracts compared to *E. coli.* From an industrial perspective, this reduces the burden of endotoxin removal in downstream processing, thereby lowering overall production costs. However, even after substantial optimization efforts – including adjustments in plasmid concentration, ribosomal binding site (RBS) sequences, and reagent formulations – these hosts produced lower yield of recombinant protein compared to *E. coli* [32].

In recent applications, *E. coli* CFES has been increasingly explored for phage production and engineering. With a highly characterized genome, *E. coli* strains have been engineered to adjust positive and negative regulatory effectors that influence gene expression, facilitating the upregulation or downregulation of desired pathways that promote the production of a desired gene. Given its efficiency and adaptability, *E. coli* remains the primary choice for producing a variety of phages, with different strains engineered for specific CFES applications, as previously documented [15]. For phage synthesis, researchers prioritize strains that enable high protein production while managing cellular stress responses, which are crucial in optimizing phage yield and stability [42]. Table 1 summarizes the hosts and phages generated from phage-tailored studies published to date.

**Table 1:** Current CFES specific for phages’ production

Year of publication	Phage bacterial host	Type of host	Cell extract preparatory host	Genetic/System modifications	Phage	Phage titer (PFU/mL)	Reference
2024	*E. coli*	Gram-negative	*E. coli* JM109	Addition of 15µL GamS (150 mM) 100 ng gDNA	M13	10^5^	Xu et al. [66]
2024	*E. coli*	Gram-negative	*E. coli* JM109 cells harboring the helper phage plasmid	Addition of 15µL GamS (150 mM) a. Phagemid b. Phagemid with the pMB1 origin of replication	M13	a. 10^8^ a. 10^9^	Xu et al. [66]
2024	*E. coli* B	Gram-negative	*E. coli* BL21-∆recBCD Rosetta2	T7 genome is re-assembled from long PCR fragments to allow for genetic manipulations in the study. “GenBank consecutive accession codes PP384393 to PP384410”.	T7 (Boca Scientific, #310025)	10^10^-10^11^	Levrier et al. [57]
2024	*E. coli* B	Gram-negative	*E. coli* BL21-∆recBCD Rosetta2		Phage T6 169 kbp GenBank: NC_054907	~ 10^8^	Levrier et al. [57]
2024	*E. coli* B^E^ [67]	Gram-negative	*E. coli* BL21-∆recBCD Rosetta2		Phage VpaE1 88 kbp GenBank: NC_027337.1	~ 10^10^	Levrier et al. [57]
2024	*Salmonella *LT2	Gram-negative	*E. coli* BL21-∆recBCD Rosetta2		Phage FelixO1 86 kbp GenBank: NC_005282	~ 10^8^	Levrier et al. [57]
2024	*Salmonella* LT2	Gram-negative	*E. coli* BL21-∆recBCD Rosetta2		Phage S16 160 kbp GenBank: NC_020416	~ 10^8^	Levrier et al. [57]
2024	*Salmonella enterica* subsp. enterica serovar Typhimurium strain LT2	Gram-negative	*E. coli*	a. Without supplemental energy mix b. With an energy mix which includes dNTPs	Phage FelixO1 86 kbp GenBank: NC_005282	a. 5.4 × 10^7^ b. ~4.0 × 10^8^	McFarlane et al. [68]
2024	*E. coli* strain B^E^	Gram-negative	*E. coli*	Without supplemental energy mix	VpaE1	7.5 × 10^10^	McFarlaneet al. [68]
2023	*E. coli* BL21 ATCC BAA-1025	Gram-negative	*E. coli* BL21 ATCC BAA-1025	Overexpression of *inf C*, OxyS, and CyaR	T7 (ATCC BAA-1025-B2)	~ 10^8^-10^9^	Brooks et al. [28]
2023	*E. coli* BL21 ATCC BAA-1025	Gram-negative	*E. coli* BL21 ATCC BAA-1025	Repression of *recC*, and *rna*	T7 ATCC BAA-1025-B2)	~ 10^7^-10^9^	Brooks et al. [28]
2022	*E. coli* K603, F+	Gram-negative	*E. coli* BL12 (DE3)	pQβ7 plasmid or Qβ (+)-RNA 3% PEG 6000 Cell extract of 24.3 mg/mL protein	Qβ Shaklee et al. [69]	~ 10^6^	Rasmussen et al. [70]
2022	*E. coli* EV36	Gram-negative	*E. coli* Rosetta 2 transformed with pAD-LyseR plasmid	Using CRISPER/Cas 9 selection and SpyTag/SpyCatcher system to attach proteins to the minor capsid protein	K1F & K1F-GFP-SpyTag	10^9^	Liyanagedera et al. [24]
2022	*E. coli* DSM 613	Gram-negative	*E. coli* Rosetta 2 (DE3)	Addition of 2µL GamS (150 µM)	T7 phage, DSM 4623	10^12^-10^11^	Emslander et al. [52]
2022	*E. coli* DSM 613	Gram-negative	*E. coli* Rosetta 2 (DE3)	Addition of 2µL GamS (150 µM)	MS2 phage, DSM 13767	10^12^-10^11^	Emslander et al. [52]
2022	*Bacillus subtilis* DSM 5547	Gram-positive	*E. coli* Rosetta 2 (DE3)	*B. subtilis* housekeeping sigma factor SigA Addition of 2µL GamS (150 µM)	Phi29 (NCBI ID: NC_011048.1) DSM 5546	10^10^	Emslander et al. [52]
2022	*Bacillus subtilis* DSM 402	Gram-positive	*E. coli* Rosetta 2 (DE3)	*B. subtilis* housekeeping sigma factor SigA Addition of 2µL GamS (150 µM)	Goe1 (vB_BsuP_- Goe1, NCBI ID NC_049975.1) DMS 101030	10^10^	Emslander et al. [52]
2022	*Yersinia pestis* EV76	Gram-negative	*E. coli* Rosetta 2 (DE3)	Addition of 2µL GamS (150 µM)	PhiA1122 (NCBI ID: NC_004777.1) [71]	10^8^	Emslander et al. [52]
2022	Enteroaggregative *E. coli* [72]	Gram-negative	*E. coli* Rosetta 2 (DE3)	Addition of 2µL GamS (150 µM)	CLB-P3 (GeneBank: OL800706)	10^10^	Emslander et al. [52]
2022	*Klebsiella pneumoniae* (Bw1)	Gram-negative	*E. coli* Rosetta 2 (DE3)	Addition of 2µL GamS (150 µM)	vB_KpS_Muc5 (GeneBank: OM687892)	10^8^	Emslander et al. [52]
2021	*E. coli*	Gram-negative	*E. coli* Rosetta 2 (DE3)	Using 5 sonication cycles and 0.5 mg/mL lysozyme	T7	Up to 10^9^	Falgenhauer et al. [53]
2021	*E. coli* B	Gram-negative	*E. coli* BL21 Rosetta 2	Addition of 3 µM of chi6 short DNA Addition of 0.1 mM dNTPs PEG8000, 3.5% (4.3 mM)	T7 (Boca Scientific)	10^13^	Garenne et al. [44]
2018	*E. coli* B	Gram-negative	*E. coli* BL21 Rosetta 2	1 nM gDNA Magnesium glutamate 4–7 mM Potassium glutamate 40–80 mM PEG800, 3% v/v	T4	10^8^	Rustad et al. [30]
2017	*E. coli* B	Gram-negative	*E. coli* BL21 Rosetta 2	Addition of 0.5 µM Chi6 short DNA 0.25 nM gDNA	T7 (Boca Scientific)	~ 10^11^	Marshall et al. [73]
2016	*E. coli* B	Gram-negative	*E. coli*	Addition of dNTPs 0.25 nM gDNA	T7 (NCBI ID: NC_001604.1)	3.35 × 10^11^	Garamella et al. [19]
2016	*E. coli* HF4714	Gram-negative	*E. coli*	5 nM gDNA	PhiX174 (GenBank: J02482.1)	1.9 × 10^12^	Garamella et al. [19]
2016	*E. coli* C-1	Gram-negative	*E. coli*	150 nM gDNA	MS2 (NCBI ID: NC_001417.2)	4.23 × 10^12^	Garamella et al. [19]
2012	*E. coli* C	Gram-negative	*E. coli* BL21 Rosetta 2	Addition of 0.5 nM dNTPS 30 nM gDNA 3.3 µM GamS	PhiX174 (New England Biolabs)	~ 10^6^	Shin et al. [74]
2012	*E. coli* B	Gram-negative	*E. coli* BL21 Rosetta 2	Addition of 0.5 nM dNTPS 1 nM gDNA 3.3 µM GamS	T7 (Boca Scientific)	~ 10^10^	Shin et al. [74]

kbp: kilobase pairs

## Reaction mixture and manipulations

In cell-free bacteriophage synthesis (CFBS), the reaction mixture is crucial in providing the necessary components to support the complex, multi-stage process of phage assembly, unlike simpler protein synthesis systems. This mixture must be optimized with the right buffering, energy sources, and essential ions, as phage assembly requires high energy and precise protein folding for infectivity. Recent advances have shown that adding energy sources such as phosphoenolpyruvate (PEP) and creatine phosphate, as well as folding chaperones, can extend reaction duration and improve protein assembly, addressing two key challenges in CFBS. Further, customizing nucleotide and cofactor concentrations and including phage-specific gene expression controls helped streamline phage genome replication and the sequential expression of structural proteins essential for building viable phage particles.

### Typical CFES reaction solution composition

During the preparation of the cell lysate, the concentration of proteins is decreased by a 20–30 factor, and genetic information (i.e., DNA and RNA) is removed from the lysate [75]. For basic *E. coli* TX-TL reactions, common components are as follows: the cell extract; the buffer system, composed of energy mix (i.e., ATP), amino acid mixture, cofactors, potassium, magnesium, and molecular crowding agents (e.g., PEG 8000); and the DNA template. Typically, the entirety of the reaction should be composed of 33% (v/v) of the cell extract and 66% of the energy buffer, amino acids, and DNA templates or plasmids [30] or 75% buffer and extract and 25% DNA [12].

Starting with the DNA template, there are several types which can be incorporated into a CFES: linear DNA (e.g., fragment of genomic DNA, a PCR-amplified segment, or synthetic DNA), plasmids, or genomic DNA (gDNA). Generally, plasmids are the preferred template due to their stability and resistance to degradation by nucleases, compared to linear DNA [73, 76]. The RecBCD complex, also called exonuclease V, is the major pathway for double-strand break repair [77] and serves as the primary nuclease responsible for degrading linear DNA in the *E. coli* CFES [78]. To avoid this issue, the cell-free extracts were prepared from an *E. coli* strain with *endA* and *recC-ptrA-recB-recD* deleted [17, 28]. However, this is not optimized for CFE in general and phage production purposes in specific. The other alternative approach relied on the fact that RecBCD stalls on DNA χ sites as part of homologous recombination [79], therefore the addition of short dsDNA sequence containing multiple χ sites stabilized linear DNA templates and enhanced the production of different proteins including T7 phage production as well [73]. Another approach would be the addition of Gam protein that binds and inhibits the RecBCD complex and was proven effective as well with phage genomes [19, 74, 78].

The concentration of the DNA template added to the system typically falls in the nanomolar range, from 0.1 nM and up to 1 nM, taken from stocks of an ideal maximum of 100 nM [5, 30, 75]. However, optimal template concentration varies based on the template size [38]. The extraction of phage gDNA can be achieved through two well-known methods: the phenol/chloroform method or viral DNA extraction kit. Whichever method used, it is generally recommended to start with a high phage titer, above 10^10^ PFU/mL to ensure proper DNA concentration by the end of the extraction [80]. Additionally, several parameters need to be checked to ensure successful expression of the phage genomes in the later steps. These include the DNA concentration using a Qubit fluorometer, the purity with a NanoDrop™ to check the A260/A280 and A260/A230 ratios, and the integrity of the extracted DNA by gel electrophoresis.

In 2024, McFarlane et al. conducted a study to produce *Salmonella* phages in CFES and compared three gDNA extraction methods including Proteinase K digestion, heat denaturation at 75 °C, and purification with commercial gDNA purification kit. Heat denaturation and the commercial kit were successful, but the Proteinase K digestion failed to release gDNA and only reduced the molecular mass of the produced band, possibly via partial digestion of phage tails but not the capsid [68].

For phage engineering, the CFES is proven successful when it comes to expressing mutant phages from phage genomes assembled in vitro from PCR-amplified fragments. The system yielded up to 10^11^ PFU/mL engineered phages within one day [57].

To assess the efficiency of the CFES, researchers tend to employ T7 phage transcriptional machinery and the *egfp* gene under the T7p14 promoter [5]. Encoding fluorescent proteins, such as enhanced green fluorescent proteins or mCherry render the system verifiable throughout the whole course of the reaction by exposing the expressed particles, respectively, to an excitation of 488 nm and emission of 512 nm and an excitation of 587 nm and emission of 610 nm [81]. For downstream protein purification purposes, other gene insertions, including a 6-histidine tag, are incorporated [5]. Laboratories have often opted for *E. coli* lysates due to its RNA polymerase which possesses the sigma factor 70 for translating genes. This is explained through the enzyme’s efficiency, versatility, reliable gene regulation, and abundance in many bacterial species [34, 50].

From an energy source perspective, the CFES lacks any means of generating or obtaining energy, in contrast to living cells. Hence, it presently has a limited operating period that is dependent on the energy system used and the mode of energy supply. However, in a cell-free preparation context, all energy resources are allocated to the intended application of expressing proteins rather than cellular self-replication [82]. In terms of the energy system used in the CFES, there are 6 main types: PANOx, PANOx-SP, cytomim, creatine phosphate (CP), PURE systems, and 3-phosphoglycerate (3-PGA) [15]. The PANOx system, first developed in 2001, utilizes cofactors that generate ATP to prolong cell-free reactions by enabling ATP regeneration from PEP without the addition of exogenous enzymes. PANOx is an acronym for phosphoenolpyruvate (PEP), amino acids, nicotinamide dinucleotide (NAD), and oxalic acid [83]. Similarly, the PANOx-SP emerged in 2003 as an improvement to the PANOx system possessing the same base cofactors with added spermidine and putrescine in a HEPES/KOH buffer [84]. This system is flexible to cost-efficient modifications such as replacing PEP with glucose, rNTPs with rNMPs, and HEPES with Bis-Tris [85]. The cytomim system is another affordable energy system created with the aim to recreate conditions found in an *E. coli * in vivo environment and provide a stable source of energy without the undesirable by-products (i.e., inorganic phosphates, pH decrease, exogenous enzymes, etc.) [85, 86]. The cytomim energy system operates on the tricarboxylic acid cycle and therefore organisms must be grown in 2xYTP-G media (or other glucose-rich broth) [84, 85]. Furthermore, the CP energy system uses CP as the main source of energy and is an alternative to PEP and acetyl phosphate (AP) systems. In an early study on the production of chloramphenicol acetyltransferase, the AP or CP system was about 2 to 2.5-fold more productive, respectively, than the PEP system [87]. The PURE system also utilizes CP; however, the process is more refined. Two mixtures are prepared: the CP energetic mixture and the His-tagged purified cell factor mixture containing the translation-transcription machinery involved in the initiation, elongation, termination, aminoacylation processes, and essential tRNA molecular species [21, 88]. This system can be bought or made in house and is capable of producing more than 100 µg/mL of GFP after 1 h of incubation. The PURE system also avoids the hydrolysis of nucleoside triphosphates as the highly purified components are free of inhibitory substances such as nucleases [21, 89–92]. However, the need for alternatives to PEP and CP emerged after a subsequent observation of non-specific degradation by phosphatases [83, 93]. In 2004, the 3-phosphoglycerate (3-PGA) system was established and demonstrated the production of the highest possible proteins yield (2.3 mg/mL) after 10 h due to its stability in *E. coli* extracts [35, 65]. Finally, as the understanding for metabolism and glycolysis in the system context grew, the use of glucose-6-phosphate became another viable option as a source of phosphate and donor for ATP regeneration [15, 30, 34]. Most CFES employ some variation of these systems and tailor them through optimization for the needs of their study and factors such as cost, pH stability, reagent availability, etc. Regardless of conditions or methods, CFES will always necessitate a source of phosphate for energy production.

Most systems follow a standard recipe with a few alterations to adapt and optimize the mixture for phage synthesis. The concentration of the used components in the reaction mixture differs, though not dramatically, between different studies. The common buffer mix is composed of 50 mM HEPES (pH 8), 1.5 mM ATP and GTP, 0.9 mM CTP and UTP, 0.2 mg/ml tRNA, 0.26 mM coenzyme A, 0.33 mM NAD, 0.75 mM cAMP, 0.068 mM folinic acid, 1.25 mM leucine and 1.5 mM for the other amino acids, 2–12 mM magnesium glutamate, 40–160 potassium glutamate, 1mM spermidine, 30 mM 3-PGA, 2% PEG8000, and either 10–15 mM maltose or 20–40 mM maltodextrin [12, 15, 30, 34]. The polyphosphate molecule hexametaphosphate has also been shown cost-efficient when coupled with maltodextrin, triggering glycolysis [15, 43, 49]. Additionally, when lactose is added to a maltodextrin-based reaction, there is an increase of protein synthesis whether from circular or linear DNA, without the addition of costly stabilizers such as GamS [43].

Previous literature has outlined details of every possible additive in CFES buffer composition and their roles in the mixture, allowing for each laboratory to optimize their cell lysates to the function of their reaction as well as choose constituents that work in a time and cost-effective fashion [15]. Furthermore, the blend and composition of canonical amino acids can either be bought or formulated in the laboratory. These 20 amino acids are dissolved in an aqueous solution, typically between a pH of 6.0 and 9.0, and for each, a concentration between 1.5 mM and 3 mM [15, 30, 75]. To improve the overall kinetics and performance of the CFES, each cell extract is supplemented with an optimized concentration of Mg-glutamate and K-glutamate in Tris-HCl buffer. The respective concentrations usually range between 4-7 mM and 0-100 mM. These ions are essential for proper transcription and translation function and can cause a great decrease in yields if the concentration intervals are exceeded [15, 30, 81]. The amino acids, ions, and DTT are often combined within this buffer.

### CFES in phage genome replication

For phage genome modifications, so far, simple approaches that mainly target changes in the CFES components were tested rather than the complicated engineering processes. The key phage directed CFES studies are listed and summarized in Table 1 and the recent case studies will be further discussed below. The genome replication and manipulation of T7 phages was investigated using the cell-free small DNA (sDNA) technique (CF-sDNA) to suppress targeted gene’s expression without modifying the genome [54]. This can be done using small RNA (sRNA); however, sRNA may be too unstable to survive in a cell-free lysate and sDNA molecules were used due to their superior stability. These antisense sDNA molecules function by targeting the ribosome binding site (RBS) at specific sequences of the mRNA strands, resulting in the downregulation of the associated proteins [54]. The success of this CF-sDNA method was first demonstrated by regulating the expression of YPet, a fluorescent protein, then of the T7 phage capsid proteins, which demonstrated reduced titers and increase in its genome replication at least 8 fold. Indeed, the CF-sDNA repression technique was proved to be promising for cell-free phage genome manipulations.

From a phage engineering angle, Emslander et al. 2022 study describes phage protein transient engineering without altering the phage genome in a CFES. The aim of the study was to modify the T7 phage’s minor capsid protein, Gp10B, by adding a polyhistidine-tag (His-tag) for affinity purification and a segment of the split luciferase enzyme (HiBit) for bioluminescent detection [52]. Since the non-genomic modification cannot be passed on to the progenies, the experimented protein level bioengineering approach offers a safe platform to systematically modify phages in response to emergent resistant organisms. In the same study, they attempted CFBS with relatedness up to order level, with the following phages and hosts: *E. coli* with MS2 and T7 phage, enteroaggregative *E. coli* with CLB-P3 (same species), *Klebsiella pneumoniae* with MUC5 (same family) and *Yersinia pestis* with PhiA1122 (same order). Results demonstrated engineered phages with the desired modifications, virulence, and high titers ranging from 10^12^ PFU/mL to 10^8^ PFU/mL. The *K. pneumoniae* and *Y. pestis* hosts produced the lowest titres and *E. coli* the highest [52]. Moreover, phages Phi29 and Goe1, targeting the Gram-positive *B. subtilis*, were successfully produced from the same *E. coli* based systems through the additional expression of the host-specific factor SigA which enabled assembly of the *B. subtilis*-targeting phages [52].

A study published in 2023 expounds on the components and mechanisms of CFBS necessary for improving yields with an engineered *E. coli* strain (BL21) [28]. Due to the conditions of CFBS, it evades the need for a propagation host, therefore the researchers preferred to make use of the endotoxin-free *E. coli* BL21 which absolves the need for additional purification steps. The research team aimed to ascertain the capacity of this strain to support phage propagation by inducing CRISPR interference (CRISPRi) and assessing how this positively or negatively impacts genes implicated in T7 propagation. Additionally, they monitored the production of sfGFP (Superfolder GFP) to confirm the activity of the T7 gDNA-dependent transcription. Results revealed that overexpression of initiation factor IF-2 (*inf*C) and the sRNAs OxyS and CyaR, as well as the repression of RecBCD (subunit RecC) increased the phage T7 CFBS by a factor of 10. The phage propagation mixture used 0.5 nM T7 gDNA and included 3.5% (w/v) PEG-8000 and 0.5 mM dNTPs [28].

More recently, the CFES was used to produce M13 phages [66]. M13 is a filamentous phage belonging to the Inoviridae with a single-stranded DNA (ssDNA) of around 6.4 kb length and a rolling circle replication mode. The impediment with this phage is that its protein coat is formed mainly by protein VIII and a few copies of proteins VII and IX, hence an unequal protein copy number is required for its assembly [94]. The initial results showed the functionality of the CFES in M13 phage production using the gDNA; however, the phage titer was much lower than in vivo methods. To improve the phage synthesis and yield in the CFES, the researchers simplified the phage genome to remove the pressure of unnecessary gene expression [95, 96]. This was done by pre synthesizing specific genes in a helper phage (HP) plasmid and transforming it into the *E. coli* JM109 host strain to synthesize the phage propagation-related genes beforehand. Then, phage production was determined by adding phagemid. This approach greatly increased the production speed and yield of phages. At 3 h, the titer plateaued at 10^8^ PFU/mL, which was significantly faster than in vivo production. Finally, a high-copy origin of replication (ori) pMB1 was added into the HP and this modification allowed the system to yield an M13 phage titer of 10^9^ PFU/mL, which is marginally less than the in vivo yield [66].

## Reaction conditions

Given that CFES currently offers a plug and play platform for on-demand tailored phage production, fine-tuning the reaction conditions is a key step to refine the end product and align with the required production scale, while ensuring overall efficiency and cost-effectiveness.

Generally, CFES reaction volumes is between 10 and 20 µL to 1 mL, and should be inside a container of appropriate size to accommodate a proportion of 1:10, to allow proper oxygenation [58]. The reaction can be done in 1.5 mL tubes, multiwell plates, semi- continuous systems, microfluidics, and liposomes. Smaller volumes under 100 µL can be placed in V-bottom multiwell plates without any shaking for aeration, while anything greater will require shaking at about 100 rpm [5, 19]. To minimize loss of volume through evaporation, these containers should be tightly closed and kept at uniform temperatures to avoid condensation. It is thus recommended to use an incubator rather than a dry block or a water bath, to ensure that the cap stays at the same temperature as the rest of the reaction solution [5].

Preparations and manipulations of stocks for reactions may include gentle vortexing (< 4,000 rpm) under 10 s, a minicentrifuge to gather the solution together after mixing, and 70% ethanol to keep the space decontaminated [5]. The temperature used for the CFES vary from 29 °C to 37 °C, with the lower temperatures most used. This depends on the proteins expressed and on whether the reaction is batch or semi-continuous. Chizzolini et al. commenced 9 µL batch CFE reactions with the PURExpress in vitro protein synthesis kit at 37 °C [92]. The use of semi-continuous system rather than batch ones has been studied and proved to work well with the CFES as it increased the yield of protein synthesis by extending the CFE lifetime using a steady nutrient supply [97–99]. In a 2016 study, they performed a semi-continuous CFE at 29–30 °C, maintaining a constant rotation (0.125 Hz) on a rotating shaft and using a 96-well dialyzer plate. The feeding solution had a similar composition to the reaction, except that they replaced the extract and plasmid with S30B buffer and water, respectively [19].

Reaction times of CFES may vary but are often within a certain range. It is somewhat surprising that the reaction time required to generate proteins (8–20 h on average) tends to be twice longer than the time for phage production, which requires generating and assembling multiple phage proteins. The reason for this is not clear. The All *E. coli* TX-TL Toolbox 2.0 paper details protocols to optimize and facilitate a wide range of experiments in synthetic biology and cell-free gene expression studies. The authors recommended cell-free expression reactions incubate 8–10 h in batch mode [19], while in the 3.0 version, they suggested up to 20 h and enhanced the CFES with increased flexibility, higher yields, and extended applications [44]. In this regard, a cell-free system for T7 phage production was engineered and optimized to achieve titers over 10^8^ PFU/mL after 20 h [28]. Moreover, Vogele et al. used antisense DNA for gene silencing in a cell-free system which enabled precise manipulation and replication of phage genomes. Their reactions for assembling phages took about 4 h and they obtained a titer of ~ 10^10^ PFU/mL [54]. Using SpyPhage, a CFE platform designed for the swift and flexible engineering of phages tailored for specific therapeutic applications, 5 h of incubation generated a maximum titer of 10^9^ PFU/mL [24]. PHEIGES, a fully cell-free system for synthesizing and selecting phages derived from engineered genomes, has shown to achieve concentrations of 10^10^-10^11^ in just 3 h of incubation [57]. Production of infectious Qβ phages, a type of *E. coli* phage with a positive single-stranded RNA genome, varied from 5 to 16 h of incubation, depending on varying factors in the conditions [69]. The Noireaux lab reported a maximum of 5 h incubation for T7 phage production yielding 10^10^ PFU/mL [74]. Similarly, the generation of personalized phages against *E. coli*, *Y. pestis*, and *K. pneumoniae* were incubated in a 13 µL cell-free reactions for 4 h at 29 °C and generated phages of titers between 10^8^ and 10^12^ PFU/mL [52]. A TX-TL reaction on T7 phage with the addition of short DNA containing χ sites (to enhance DNA stability) ran for 12 h at 29 °C and yielded 10^11^ PFU/mL [73]. While in a cell-free synthesis of T4 phage, the reaction was incubated for 10–12 h, and most phages were produced between the first 2–4 h before reaching a plateau at the 6–10 h mark [30].

## Current limitations of CFES

Cell-free systems are valuable tools for producing proteins, but they do have limitations that must be considered. Below are the key limitations of the CFES, along with mitigation strategies to address these challenges.

The main limitation of the CFES is the depletion of necessary components and energy over time and the production and accumulation of inhibitory by-products, which leads to a loss of protein synthesis efficiency. However, this mainly happens in batch formats of the system and can be overcome using continuous-flow and continuous-exchange CFPS reactors. Those reactors supply reactants and remove by-products, which in turn prolongs the reaction duration and increase the protein yields [100, 101]. The second limitation is the high cost of the commercially available CFES if intended for large scale protein production [40]. This can be addressed through preparation of in-house cell extracts, optimizing the reaction mixture components, and tailoring the reaction time and temperature based on the intended product, which shall lower the overall cost of the system when streamlined [41]. Another consideration is the capacity of the system to accommodate protein folding and post-translational modifications (PTMs), such as glycosylation or phosphorylation. This is specifically challenging in *E. coli*-based systems, owing to the lack of endogenous glycosylation machinery and the limited number of PTMs possible in bacteria when compared to eukaryotes [102]. However, it has already been proven possible through the supplementation of glycosylation machinery from a glyco-optimized chassis host strain [103]. Yet, this is an area that needs further studies and optimization steps.

## Conclusions

Cell-free expression is a revolutionary technique that offers a range of applications, both in research and industry [104]. The system was initially developed for protein production, specifically the difficult-to-express proteins, toxic proteins, or proteins with unnatural amino acids. This allows for versatile downstream applications, based on the end-in-mind target, such as high-throughput screening of enzyme variants, small molecule inhibitors for drug discovery purposes, or the production of viral protein antigens for vaccine development [105, 106]. The system is also used in biosensing applications to detect specific molecules, or environmental toxins, by producing a measurable output in response to the target [107]. The application of this technology for phage production was first described 12 years ago and is constantly improving [74].

The cornerstone of the cell-free system is the transcriptional and translational cell machinery; thus, streamlining the crude extract preparation is imperative to remove inter- and intra-laboratory variabilities and transition the system from a laboratory context to industry. Although the protocols for cell extract production are well established, there are several factors to be considered to reduce batch-to-batch variability. The growth time for cell extract harvesting is a key variable in cell extract preparation. This step depends mainly on measuring the absorbance at 600 nm, given the availability of spectrophotometers in almost every laboratory. However, to avoid interlaboratory variability that might rise from differences in culture media constituents or spectrophotometers differences, measuring actual cell density would be more robust. This can be achieved via in situ turbidity sensors [108]. Additionally, online predictive growth models for shake flasks can be used when testing a new strain for cell extract production for predictions of biomass at certain time points, and thus setting a time for the culture harvest [109]. Another effective approach may involve automating the preparation of extracts, although this will require scaling up to offset the resource expenditure.

As for the cell lysis step, phage production targeting studies have opted for variable methods, depending greatly on the organism used for cell extract preparation. Given that *E. coli* is the only organism tested to date for the system, the availability of equipment would be the limiting factor here. Interest in the system for phage production is increasing; thus, advances involving other organisms could be seen soon which will require variability in the lysis step. It is also noteworthy that the sequence of post lysis steps, which is clarification followed by runoff, is to be carefully followed and considered for an active cell extract production.

To ensure a smooth workflow of cell-free reactions, some recommendations during the buffer preparation step include making large stock solutions of all mixtures, such as the S30 buffer and amino acids, and leaving the quickly degradable reagents (i.e., energy mix) for the week of the experiment. Similarly, silica beads can be added to the reaction volumes to further stabilize the reagents within the lysate mixture and cancel out the possibility of denaturation. As previously mentioned, pre-formulated liquid amino acid mixtures can be bought. Unfortunately, this option is costly and can be difficult to customize for specific experiments, especially if the research includes use of non-canonical amino acids. Purchasing these in powder form is a more versatile and economical solution.

It is essential to consider every component of the reaction mix to reflect the kind of product being produced and adjust the reaction conditions that will be used. One may start with the general preparation and gradually tweak some elements based on the readily available equipment.

The other important aspect to be considered is the diversity of phages this system can handle. Based on the current streamlined *E. coli* cell extract, the naturally available machinery supports T7 gene expression as it is a natural host for T7 phages. The process also capitalizes on the cellular machinery (i.e., ribosomes, tRNAs, transcription factors) and native components that are already optimized to handle the codon usage and processing of T7 proteins, ensuring that the cell-free system runs smoothly without the need for extensive modifications. Recently, the system has been proven efficient beyond *E. coli* phages when specific transcription factors or additional host factors are added and expressed. Further improvement of the system can be accomplished by modifying the genetic background of the strain used to generate the cell lysate to alter the transcription and translation machinery derived from it. This is a growing area of research which shows a lot of potential for producing non-canonical phages, such as those that infect anaerobic bacteria.

In conclusion, the CFES is a revolutionary platform for producing complex structures like phages which requires coordinated expression of many genes. The system offers swift phage generation without the need for living bacterial cultures. This is particularly useful in synthetic biology, where researchers can rapidly modify phage genomes and express them in vitro, making it easier to efficiently prototype phages that have been developed for biotechnological, medicinal, or diagnostic applications.

## Data Availability

No datasets were generated or analysed during the current study.

## Abbreviations

× g: Relative centrifugal force (RCF) expressed in units of gravity (times gravity or × g)
2xYTP: Yeast extract tryptone/phosphates
3-PGA: 3-phosphoglyceric acid
CF-sDNA: Cell-free small DNA
CFBS: Cell-free bacteriophage synthesis
CFE: Cell-free expression
CFES: Cell-free expression systems
CFGE: Cell-free gene expression
CFPS: Cell-free protein synthesis
CP: Creatine phosphate
DTT: Dithiothreitol
gDNA: Genomic DNA
GFP: Green fluorescent protein
GOI: Gene of interest
HP: Helper phage
J: Joules
Kbp: Kilobase pairs
KHz: Kilohertz
LAS: Lysozyme-assisted sonication
NAD: Nicotinamide dinucleotide
PEG: Polyethylene glycol
PEP: Phosphoenolpyruvate
PFU: Plaque forming units
PSI: Pounds per square inch
PURE: Protein synthesis using recombinant elements
RBS: Ribosome binding site
RPM: Revolutions per minute
TX-TL: Transcription-translation
